# Influence of Sex-Mismatch on Prognosis After Heart Transplantation

**DOI:** 10.3389/fcvm.2021.617062

**Published:** 2021-03-25

**Authors:** Ana Ayesta

**Affiliations:** Heart Area, Hospital Universitario Central de Asturias, Oviedo, Spain

**Keywords:** sex-mismatch, transplantation, prognosis, size-mismatch, rejection

## Abstract

The influence of donor and recipient sex on prognosis after heart transplantation has been analyzed in single, multi-center studies, and international registries. In most of them, sex-mismatch was identified as a risk factor for the worst prognosis, especially in men recipients of female heart. This could be attributed to physiological differences between women and men, differences in complications rates after heart transplantation (rejection, cardiovascular allograft vasculopathy, and primary graft failure), and pulmonary hypertension of the recipient. Confounding variables as age, urgent transplantation, and size-mismatch should also be considered. When allocating a graft, sex-mismatch should be considered but its influence in long-term survival should be further explored.

## Introduction

Heart failure (HF) is a clinical syndrome appearing in the final pathway of heart disease. It affects 1–2% of the adult population and it increases with age. The development of symptoms leads to morbidity, mortality, and poor quality of life. It has a poor prognosis, and heart transplantation (HT) is the treatment of choice in selected patients (1). When allocating a graft, donor, and recipient characteristics should be considered (2). Among them, the influence of donor/recipient sex-mismatch on prognosis has been broadly discussed. In this manuscript, we will address this issue and will try to figure out the mechanisms underlying this relationship.

## State of the Art: Donor/Recipient Sex-Mismatch Influence on Heart Transplantation Prognosis

### Influence on Early and Long-Term Survival

Initially, donor, and recipient sex influence on mortality were analyzed separately (3–9). After heterogeneous results, the influence of donor/recipient sex-mismatch was analyzed. In 1998 two studies found an influence on early mortality (10) and worst annual survival (11), due to lower survival in the female donor to male recipient (F/M) group, attributed to size-mismatch. Later, several studies confirmed this relation. In 2011, a single-center study with 857 patients did not show worse survival of F/M group compared to male donor to female recipient (M/F) group, although a trend in early mortality was suggested and better survival in recipients without mismatched heart was shown (12). Other studies reported significantly worst survival of F/M group in early stages after HT (13–15), while other authors related sex-mismatch with mortality regardless of the recipient sex (10, 16–18). However, in Bello et al. (16) sex matched pairing conferred a survival benefict, and M/F combination had worst survival. On the contrary, others failed to relate sex-mismatch with poorer prognosis (19–24). In this sense, De Santo et al. (19) found no differences in one and three-year cumulative survival between sex-mismatch and sex-match patients in a cohort with 99 patients. Jalowiec et al. (20) found in a multicentric analysis of 347 patients no significant differences in early survival (30-days and 1-year survival) between sex-mismatch and sex-matched patients. Tsao et al. (21) and Yamani et al. (22) also did not find differences in survival between 4 groups created according to donor/recipient sex. In 2014, Correia et al. published the results of the analysis of 200 male recipients in a Portuguese center. They did not find higher mortality in sex-mismatch group than in sex-matched group. The authors reported selection bias, as recipients of mismatched hearts had lower pulmonary gradient and lower systolic pulmonary pressure (24). The results of the Spanish Heart Transplantation Registry published in 2014 included 4,625 patients and found an influence of sex-mismatch on early mortality only in male recipients and mainly in those with pulmonary gradient >13mmHg (25).

The results of the analysis of large registries, expected to be more accurate and reliable, also reported heterogeneous results (26–32). In 2002, Zeier et al. (29) found higher mortality of F/M group analyzing the Collaborative Transplant Study database. The United Network for Organ Sharing (UNOS) database analysis published in 2009 (28) compared 4 groups, based on the combination of donor and recipient sex, and showed a lower survival at 5 years in the F/M group and greater survival in the male to male (M/M) group. A later analysis of this same database (31) found that survival differences associated with sex-mismatch were modified by differences in predicted heart mass (PHM) by a mathematical model. In a retrospective analysis of 31,634 patients, the authors found that a difference of 10 to 15% in PHM (undersized heart) between donor and recipient resulted in higher risk. In fact, when adjusting by PHM, they showed higher mortality in M/F group. The results of the International Society for Heart and Lung Transplantation (ISHLT) have also been analyzed several times (26, 30, 32). In 2012 (30) an increase in mortality in F/M was reported compared to M/M, influenced by early mortality. Later, Kackmarek et al. (26) analyzed 67,855 transplanted patients and found the worst annual survival rates in F/M group. The most recent analysis included 52,455 patients (32) and found that sex-mismatch increased mortality independently of weight match. The results of the University of Alabama – Cardiac Transplant Research database (CTRD), previously published, had found an interaction between sex, weight mismatch, and survival, especially in F/M. However, these differences were not observed when the weight mismatch was minimum (27).

A meta-analysis addressing sex-mismatch influence on one-year survival has been recently published (33). After an initial search, 556 articles were found, and 45 articles were selected for full-text assessment. Finally, only 10 articles were included for data extraction and quantitative synthesis. 76,175 patients were analyzed. In male recipients, sex-mismatch was related with increased one-year mortality (21.2 vs. 16.6%; OR = 1.38, 95% CI 1.31–1.44, *p* < 0.001). On the contrary in female recipient sex-mismatch was not a risk factor for one-year mortality (18.2 vs. 18.6%; OR = 0.93, 95% CI = 0.85–1.00, *p* = 0.06). The main limitations of this meta-analysis are the strong influence of the largest registry included in the results (26), the inability to determine the real influence of confounding factors and to determine the influence of early complications on long-term survival. However, it is the first meta-analysis on this field with studies of low bias, and the population included is representative of the HT population.

### Influence on Rejection

The influence of sex-mismatch on rejection is unclear. Differences in the endocrine and immune system could lead to different adaptations to sex-mismatched heart (34). Women have a greater immune response (6, 35, 36) that leads to higher levels of immunoglobulins and autoimmune diseases (37) and are supposed to have higher rates of rejection (6–9, 38). In 1998, Prendergast et al. (11) found higher rates of acute rejection in recipients with a sex-mismatched heart, as also did Aliabadi et al. (23) in 2011. In 2012, Jalowiec et al. (20) reported higher rejection rates in M/F as had been previously published (39) and related lower survival to higher steroids requirements in the early post-transplant period. Patel et al. (40) reported, in a group of 1,299 patients, higher antibody-mediated rejection in M/F, but a recently published study found a higher risk in female recipients regardless of sex-mismatch (41). On the contrary, Bryan et al. (42) reported lower rejection rates in recipients of male hearts, mainly due to lower rates of the M/M group compared to the F/M group.

### Influence on Cardiac Allograft Vasculopathy

The influence of sex-mismatch on cardiac allograft vasculopathy (CAV) has also been studied with heterogeneous results. A higher risk of CAV in F/M group was reported in different studies (38, 43). Whether these results were attributed to sex-mismatch, female donor or male recipient is not clear (44–46). Other studies showed this relationship regardless of the combination (23) or in the F/F group (22). Eifert et al. (13) failed in 2012 to show this relation. Immunological or size-mismatch could be the reason underlying this association (38, 43).

### Influence on Primary Graft Failure

Primary graft failure (PGF) is an impairment of the transplanted heart that occurs in the first 24 h after transplantation (47). It is the main cause of death in the early post-transplant period with up to 22% mortality (48). In an analysis of the Spanish Registry of Cardiac Transplantation (25) an increase in mortality in F/M in the first 30 days was found, but PGF was related to female donors, as previously noted (49) but not with sex-mismatch. However, some studies found a relation of PGF with sex-mismatch in male recipients (50–52), although Young et al. (51) found this was particularly important when the size exceeded 30%.

In Table 1 we present a summary of the main studies that show the influence of sex-mismatch on higher rates of mortality, rejection, CAV, and PGF.

**Table 1:** Summary of the main studies showing the influence of sex-mismatch on higher rates of mortality, rejection, cardiovascular allograft vasculopathy, and primary graft failure.

**Reference**	**Type of study**	**Number of patients**	**Results**
**Sex-mismatch influences on survival**			
Al-Khaldi et al. (15)	Single-center	869	- Recipient of female heart had worst survival (depending on donor/recipient age).
Ayesta et al. (33)	Meta-analysis	76,175	- Sex-mismatch affected 1-year survival in male recipients but not in female recipients.
Bello et al. (16)	Multicenter	3,316	- M/F was related with worst survival.
Eiffert et al. (13)	Single-center	1,000	- Multivariate analysis showed that F/F was a long-term survival predictor.
Kackzmarek et al. (26)	Multicenter (ISHLT Registry)	67,855	- F/M worst long-term survival.
Kittleson et al. (12)	Single-center	857	- Best survival in patients with sex-matched heart. - 5-year actuarial survival worst in F/M.
Khush et al. (30)	Multicenter (ISHLT Registry)	60,584	- F/M had higher risk of mortality.
Kirsch et al. (10)	Single-center	234	- Influence of sex-mismatch on early mortality.
Martínez-Sellés et al. (25)	Multicenter (Spanish Society of Cardiology Registry)	4,625	- F/M had higher early mortality, especially in those recipients with pulmonary gradient >13 mmHg.
Prendergast et al. (11)	Single-center	174	- F/M had worst annual survival.
Reed et al. (31)	Multicenter (UNOS Registry)	31,634	- M/F had worst 1 and 5-year survival.
Schelechta et al. (18)	Multicenter	609	- Sex-mismatch recipients had worst 3 and 5-year survival.
Stehlik et al. (27)	Multicenter (CTRD database)	7,321	- In F/M, older recipients and those higher size-mismatch had worst survival.
Weiss et al. (28)	Multicenter (UNOS Registry)	18,240	- F/M had worst 5-year survival - Multivariate: higher mortality in F/M vs. M/M.
Welp et al. (14)	Single-center	236	- F/M had worst survival.
Zeier et al. (29)	Multicenter	25,432	- Worst actuarial survival in F/M.
**Sex-mismatch influences on rejection rates**			
Aliabadi et al. (23)	Single-center	1,079	- Mismatch recipients had higher rates of acute rejection.
Bryan et al. (42)	Multicenter	279	- F/M vs. M/M had higher rates of rejection. - Female donor was related with higher risk of rejection.
Jalowiec et al. (20)	Multicenter	347	- M/F had higher rates of acute rejection.
Keogh et al. (39)	Single-center	313	- M/F had higher rates of acute rejection the first 3-months.
Patel et al. (40)	Single-center	1,299	- M/F had higher rates of antibody-mediated rejection.
Prendergast et al. (11)	Single-center	174	- Mismatch recipients had higher rates of acute rejection.
**Sex-mismatch influences on cardiovascular allograft vasculopathy rates**			
Aliabadi et al. (23)	Single-center	1,079	- Mismatch recipients had higher rates of CAV
Mehra et al. (43)	Single-center	36	- F/M was the combination with higher risk of CAV using intravascular ultrasound.
Sharples et al. (38)	Single-center	323	- F/M was the combination with higher risk of CAV.
**Sex-mismatch influences on primary graft failure rates**			
Russo et al. (50)	Multicenter (UNOS Registry)	16,716	- F/M was associated with higher risk of PGF.
Singh et al. (52)	Multicenter	450	- F/M was associated with higher risk of PGF.

*UNOS, United Network for Organ Sharing; CAV, Cardiac Allograft Vasculopathy; CTRD, Cardiac Transplant Research Database; F/F, female donor and female recipient group; F/M, female donor and male recipient group; ISHLT, International Society for Heart and Lung Transplantation; PGF, Primary Graft Failure; M/M, male donor and male recipient group; M/F, male donor and female recipient group*.

## Discussion

Different analysis on sex-mismatch influence on prognosis have shown different results. Some of them found the worst survival in F/M group (11–15, 25–29, 33), while others did not. How sex-mismatch could influence on mortality is still unknown. Hypothetically, it could be due to anatomic, immune, hormone, and genetic differences between women and men. Also, differences in donor and recipient age and the emergency of the transplant could be involved. Most importantly, size-mismatch between donor and recipient and pulmonary hypertension of the recipient could be the main factors underlying this relationship and are currently being studied. The heterogeneous results in the influence on CAV and PGF are probably due to different definitions until consensus was reached.

### Anatomic and Physiological Differences

Anatomic and functional differences between women and men's hearts lead to different abilities to adapt to different hemodynamic situations (53–56). Also, in transplanted women with previous male pregnancies, the presence of male cells could better explain the ability of women to adapt to a sex-mismatched heart (57). On the contrary, differences in endocrine and immune system could increase rejection in women (34–37). Advanced donor age is also related to mortality, mainly the first year after HT (58). In some studies, female donors older than male could be the reason under the worst survival of the F/M group (15, 18, 19, 24–26). However, some studies specifically addressed failed to show an interaction between age and sex-mismatch (15, 19, 22, 24–26). However, Al-Khaldi et al. (15) found an interaction between age and donor/recipient sex. Female recipients (younger) had no impact on multivariate analysis and the M/M group was the one with the best one-year survival. This confirmed the previously published data from the UNOS registry that showed that recipient <55 years-old and donor <30 years-old had the best long-term survival (59).

### Urgent Transplant

The analysis of the UNOS Registry published in 2009 (28) showed higher mortality in F/M only valid for those transplanted in maximum urgency. A previous analysis published in Spain (60) had also shown higher mortality in the F/M, due to the higher rates of urgent transplant.

### Undersizing Effect and Pulmonary Hypertension

The most currently discussed reason underlying the relation between sex-mismatch and survival is the “under-sizing” effect. A smaller female heart would not be able to keep the cardiac output required by a man, resulting in immediate right ventricular failure (61). The use of different cardiac size measures has attempted to minimize the effect of sex-mismatch by reducing size-mismatch. However, it is still not clear that sex-mismatch influence on prognosis is totally due to size-mismatch.

An analysis of the Spanish Registry of Heart transplantation (25) showed that sex-mismatch increased mortality only in men with pulmonary hypertension the first month after HT. However, there were no significant differences in weight relationship between donor and recipient in M/M vs. F/M. In the same way, the most recent analysis of ISHLT database (32) found that sex-mismatch increased mortality independently of weight match. They analyzed 52,455 transplants between 1994 and 2013 and defined three subgroups according to BMI: underweight, non-obese, and obese. Inappropriate weight match, defined as donor weight <70% of the recipient's weight, was associated with 30-day mortality and cumulative mortality. F/M and M/F had higher rates of cumulative mortality compared with sex-matched patients but increased early mortality only in F/M. They found no interaction between inappropriate weight match and sex-mismatch, which would be expected if size differences were the main reason for increased mortality in this group. Previous analysis of the ISHLT database (26) had focused on donor and recipient body mass index (BMI). They suggested an “undersizing effect” due to F/M worse results after correction of weight and height and an “oversizing effect” with better short-term results in M/F, especially when the recipient had high pulmonary pressures. Other analysis of this same database (30) adjusted the results based on weight mismatch, using three different parameters: donor and recipient weight, donor and recipient weight difference, and weight ratio of the recipient regarding donor weight. They found worse survival in F/M, but they did not find an interaction of the difference in weight in this survival. UNOS data published in 2009 (28) studied BMI ratio and body surface area (BSA) ratio between donors and recipients, finding a quite precise adjustment, probably due to a deliberate move to allocate the graft adjusting by cardiac size. Other studies were consistent with this adjustment and showed no difference among the four groups in donor/recipient BSA ratio (15, 18, 19).

However, a poor correlation between weight and heart size was shown, questioning the suitability of the measures used so far (31). Reed et al. (31) studied a new way of assessing this relationship with a mathematical formula. They conducted a retrospective study of 31,634 patients included in the UNOS registry, identifying undersizing pairs with increased risk. The formula calculated the PHM combining the predicted left ventricular and right ventricular cardiac mass. They found that a difference of 10–15% (undersized heart) resulted in a higher risk of mortality. In the adjusted analysis, the risk attributed to sex-mismatch in F/M disappeared and higher mortality was observed in M/F. These results would agree with the theory that cardiac size-mismatch is interacting with the worst survival in F/M. A most recent analysis of the UNOS registry (19,168 recipients between 2007 and 2016) assessed the ability of 5 size match metrics: PHM, weight, height, BMI, and BSA to predict 1-year mortality after HT (62). They found that PHM is the optimal donor-recipient size for the prediction of mortality. The increased mortality associated with donor-recipient PHM undersizing below 0.86 persisted after adjusting for other factors affecting mortality, including sex-mismatch (62). The authors analyzed the role of sex-mismatch and PHM in heart offer turndown from donor size/weight. Most of them were F/M and 17% of them would be acceptable using the PHM cut off. F/M did not have an increased risk of death. The thirty-sixth adult heart transplantation report of the ISHLT published in 2019 addressed this issue (63). The authors analyzed donor-recipient size match based on PHM. They found that most of donor-recipients with weight match ≤30% had an acceptable PHM of <20 to >20%, which may lead to an increase in the use of hearts. The Pearson correlation coefficient (R) for weight mismatch compared to PHM mismatch was moderate-strong. They also analyzed donor-recipient PHM match according to sex match. F/M tended to be undersized and M/F tended to be oversized. They concluded that differences in size matching may be a part of mortality differences seen in different sex-mismatch combinations. Donor-recipient size match by PHM was identified as a significant predictor of 1- and 5-year mortality after heart transplant (for both recipients of undersized and oversized donors). A recent analysis of the OPTN/UNOS Registry (64) analyzed 3,788 F/M from 2005 to 2018. They demonstrated that increasing donor BMI relative to recipient BMI up to 1.5 was associated with improved survival. They speculated that BMI difference may be useful as a surrogate for PHM difference (due to the complexity of PHM) and might help mitigate the impact of sex-mismatch in heart transplantation.

In patients with pulmonary hypertension, it is common practice to oversize donor hearts to prevent post-operative right ventricular failure. A recently published studied analyzed patients in the UNOS Registry (65) with moderate pulmonary hypertension. They found no benefit to oversizing donors. The unadjusted 1-year mortality was significantly higher for F/M compared with M/M but after propensity matching, there was no difference in mortality between female and male donors at 90 days and 1 year. However, a higher risk for 1-year mortality persisted among M/F in comparison with M/M. Also, there might be an interaction between weight difference, age, and recipient sex. A previous analysis of the CTRD had found an interaction between weight difference, age, and recipient sex, with higher one-year mortality in F/M with an older organ (more than 40 years) and a 30% weight difference (27). A single-center Portuguese study (24) showed the same survival in those patients with sex-mismatch due to a good selection of grafts based on cardiac size in those patients with high transpulmonary gradient. However, it is a single-center and small sample study so their results cannot be considered superior to those observed on large international bases.

The influence of donor/recipient sex-mismatch on survival after HT is still not clear and the reasons underlying are still under debate. Adjusting size-mismatch may help to improve results but there are still some other factors that should be clarified. Further studies, especially prospective ones, would be necessary to improve survival and allocate the best graft in this era with scarcity of organs.

## Conclusion

The influence of sex-mismatch on prognosis after HT has been broadly studied. In brief, a worst survival of male recipients receiving female heart was noted. However, new evidence shows that the optimization of cardiac size match between donor and recipient with adequate measures could modify the effect of sex-mismatch.

## Author Contributions

The author confirms being the sole contributor of this work and has approved it for publication.

## Conflict of Interest

The author declares that the research was conducted in the absence of any commercial or financial relationships that could be construed as a potential conflict of interest.
